# Supplementary material for: An Italian Survey and Focus Groups on Fibromyalgia Impairment: Impact on Work and Possible Reasonable Accommodations
Source: Healthcare (Basel). 2024 Jan 16;12(2):216. doi: 10.3390/healthcare12020216 (PMC10815387; doi:10.3390/healthcare12020216)
Supplement: Supplementary file 1 [file healthcare-12-00216-s001.zip › healthcare-2760246-supplementary.pdf]

Supplementary materials

Box S1. Survey-questionnaire (in original language).

1. *Genere*
    a. Maschio
    b. Femmina
2. *Età (inserire l'età in formato numerico)*
3. *In quale regione abiti? (elenco a discesa)*
4. *Con chi vivi?*
    a. Da solo
    b. Con altre persone
5. *Da quanti anni ti hanno diagnosticato la Fibromialgia?*
    a. Meno di un anno
    b. Da uno a cinque anni
    c. Da cinque a dieci anni
    d. Oltre 10 anni
6. *Attualmente lavori?*
    a. Si
    b. No
7. *Da quanto tempo non lavori?*
    a. Da meno di un anno
    b. Da meno di cinque anni
    c. Da meno di dieci anni
    d. Da oltre dieci anni
    e. Non ho mai lavorato
8. *Che tipologia di lavoro svolgi o svolgevi?*
    a. Impiegato
    b. Insegnante
    c. Operaio
    d. Operatore sanitario
    e. Libero professionista
    f. Altro
9. *Indica quale affermazione rispecchia meglio il tuo stato d'animo rispetto al lavoro:*
    a. vado/andavo volentieri a lavoro, ne sono/ero molto/abbastanza soddisfatto
    b. lavoro/lavoravo volentieri, ma ho/avevo alcuni problemi rilevanti
    c. temo/temevo di poter perdere il mio posto di lavoro
    d. non vado/andavo volentieri a lavoro
10. *Le difficoltà sul lavoro causate dalla Fibromialgia dipendono da: (risposta multipla)*
    a. Ansia
    b. Relazioni interpersonali negative
    c. scarsa lucidità causata dall'assunzione delle medicine
    d. Senso di tristezza
    e. Sintomi di stanchezza cronica
    f. Variabilità del dolore
    g. Variabilità dell'umore
11. *Quando ho un problema/una difficoltà legata alla Fibromialgia o ai farmaci che prendo per i suoi sintomi preferisco non parlarne*
    a. Si
    b. No
12. *Quando ho un problema/una difficoltà legata alla Fibromialgia o ai farmaci che prendo per i suoi sintomi so a chi rivolgermi nel lavoro per avere un aiuto/supporto*
    a. Si

b. No

13. *Quando ho un problema/una difficoltà legata alla Fibromialgia o ai farmaci che prendo per i suoi sintomi sento molta solidarietà tra i colleghi attorno a me*
    a. Si
    b. No
14. *Quando ho un problema/una difficoltà legata alla Fibromialgia o ai farmaci che prendo per i suoi sintomi il medico competente/l'RLS sono un riferimento importante*
    a. Si
    b. No
15. *Quando ho un problema/una difficoltà legata alla Fibromialgia o ai farmaci che prendo per i suoi sintomi i sindacati aziendali sono un riferimento importante*
    a. Si
    b. No
16. *Quando ho un problema/una difficoltà legata alla Fibromialgia o ai farmaci che prendo per i suoi sintomi il datore di lavoro/responsabile personale/caporeparto sono un riferimento importante*
    a. Si
    b. No
17. *Quando ho un problema/una difficoltà legata alla Fibromialgia generalmente le persone nel mio ambiente di lavoro conoscono la Fibromialgia e cosa comporta*
    a. Si
    b. No
18. *Quando ho un problema/una difficoltà legata alla Fibromialgia generalmente mi credono quando riferisco i problemi che ho*
    a. Si
    b. No
19. *Come valuti le tue relazioni con i tuoi responsabili o con i datori di lavoro? (risposta multipla)*
    a. Ho la loro solidarietà
    b. Comprendono il mio stato di salute
    c. Non vengo coinvolto/a
    d. Vengo svalutato/a
    e. Ci sono stati degli episodi apertamente ostili
20. *Come valuti le tue relazioni con i colleghi? (risposta multipla)*
    a. Ho la loro solidarietà
    b. Comprendono il mio stato di salute
    c. Non vengo coinvolto/a
    d. Vengo svalutato/a
    e. Ci sono stati degli episodi apertamente ostili
21. *Hai trovato difficoltà legate all'organizzazione del lavoro, alla ripartizione dei compiti o alla mansione assegnata?*
    a. Mai avuto difficoltà di questo tipo legate alla Fibromialgia
    b. Una sola volta e l'ho risolta
    c. Una sola volta e non l'ho risolta
    d. Più volte e le ho sempre o quasi sempre risolte
    e. Più volte e non le ho mai o quasi mai risolte
22. *Hai avuto difficoltà legate agli orari (es. entrata/uscita, accesso al part time), alla presenza (es. assenze ripetute per malattia) e ritmi di lavoro (es. pause)?*
    a. Mai avuto difficoltà di questo tipo legate alla Fibromialgia
    b. Una sola volta e l'ho risolta
    c. Una sola volta e non l'ho risolta
    d. Più volte e le ho sempre o quasi sempre risolte
    e. Più volte e non le ho mai o quasi mai risolte

23. *Hai trovato difficoltà legate all'ambiente fisico, ai locali, alle strutture (es. lunghi tratti da percorrere a piedi, scale, luminosità)?*
    a. Mai avuto difficoltà di questo tipo legate alla Fibromialgia
    b. Una sola volta e l'ho risolta
    c. Una sola volta e non l'ho risolta
    d. Più volte e le ho sempre o quasi sempre risolte
    e. Più volte e non le ho mai o quasi mai risolte
24. *Hai trovato difficoltà legate alla postazione o alla seduta (es. in relazione all'ergonomia)?*
    a. Mai avuto difficoltà di questo tipo legate alla Fibromialgia
    b. Una sola volta e l'ho risolta
    c. Una sola volta e non l'ho risolta
    d. Più volte e le ho sempre o quasi sempre risolte
    e. Più volte e non le ho mai o quasi mai risolte
25. *Hai trovato difficoltà legate alle regole o a prassi aziendali?*
    a. Mai avuto difficoltà di questo tipo legate alla Fibromialgia
    b. Una sola volta e l'ho risolta
    c. Una sola volta e non l'ho risolta
    d. Più volte e le ho sempre o quasi sempre risolte
    e. Più volte e non le ho mai o quasi mai risolte
26. *Hai trovato difficoltà legate all'attrezzatura o alle tecnologie utilizzate nel lavoro?*
    a. Mai avuto difficoltà di questo tipo legate alla Fibromialgia
    b. Una sola volta e l'ho risolta
    c. Una sola volta e non l'ho risolta
    d. Più volte e le ho sempre o quasi sempre risolte
    e. Più volte e non le ho mai o quasi mai risolte
27. *Hai trovato difficoltà ad avere una promozione o migliorare l'inquadramento?*
    a. Mai avuto difficoltà di questo tipo legate alla Fibromialgia
    b. Una sola volta e l'ho risolta
    c. Una sola volta e non l'ho risolta
    d. Più volte e le ho sempre o quasi sempre risolte
    e. Più volte e non le ho mai o quasi mai risolte
28. *Quando hai un problema sul lavoro, dovuto alla Fibromialgia, quali aspetti potrebbero aiutarti? (risposta multipla)*
    a. Essere creduto
    b. Cambiamento di condizioni ambientali fisiche
    c. Cambiamento di mansione
    d. Cambiamento di orario
    e. Modifiche procedurali e organizzative
    f. Altro
29. *Se puoi, indica l'elemento che nella tua esperienza è stato più FACILITANTE nel risolvere le tue problematiche relative alla Fibromialgia, in qualunque ambito ma facendo esempi concreti. (risposta aperta)*
30. *Quando hai un problema sul lavoro, dovuto alla Fibromialgia, quali aspetti sono di maggiore ostacolo? (risposta multipla)*
    a. Non essere creduto
    b. Ambiente fisico
    c. Incompatibilità dell'assunzione dei farmaci con guida/uso di apparecchiature
    d. Tipologia delle mansioni
    e. Orari di lavoro
    f. Procedure e organizzazione del lavoro
    g. Altro
31. *Se puoi, indica l'elemento che nella tua esperienza è stato più OSTACOLANTE, in qualunque ambito ma facendo esempi concreti. (risposta aperta)*

Box S2. Complete list of questions used in the FG study.

Icebreaker question
Each participant was invited to tell a real experience and an invented one. The other participants then had to guess which of the two experiences was the invented one.

Transition question (after a brief presentation of the survey preliminary results)
"How coherent your experience was with the survey findings?".
Key questions

i. "How do you think it would be possible to overcome the obstacle of not being believed by your colleagues or supervisors/employers in the workplace?", "If one of you was able to overcome the issue, how did you do it? Which strategies and facilitators?"
ii. "How do you think workplaces could be improved?", "Which obstacles or barriers would you like to remove?", "Which supports or facilitators would you like to introduce?".

Table S1. Consolidated criteria for reporting qualitative studies (COREQ): 32-item checklist.

**No.**	**Item**	**Description**	**Section #**
**Domain 1: Research team and reflexivity**			
Personal characteristics			
1.	Interviewer/facilitator	*Which author/s conducted the interview or focus group?* MT	Materials and Methods/Focus Group Study/Data collection
2.	Credentials	*What were the researcher's credentials?*<br>MT is MSc in Clinical Psychology and Cognitive-Behavioral Psychotherapist with expertise in the treatment of Fibromyalgia (FM) patients.	Title page
3.	Occupation	*What was their occupation at the time of the study?*<br>Researcher in psychological and epidemiological aspects of chronic pain and FM at the ISAL Foundation.	/
4.	Gender	*Was the researcher male or female*? Male	/
5.	Experience and training	*What experience or training did the researcher have?*	/

		MT received a specific university training in Focus Group (FG) conduction.	
Relationship with participants			
6.	Relationship established	*Was a relationship established prior to study commencement?*<br>FM individuals had no prior relationship with the interviewer before the FGs.	/
7.	Participant knowledge of the interviewer	*What did the participants know about the researcher? E.g. Personal goals, reasons for doing the research*<br>The interviewers were introduced to participants just before the FGs started.	/
8.	Interviewer characteristics	*What characteristics were reported about the interviewer/facilitator? E.g. Bias, assumptions, reasons and interests in the research topic*<br>Participants were informed about the role of the interviewer in the ISAL Foundation and on aims and scope of the research.	/
**Domain 2: Study design**			
Theoretical framework			
9.	Methodological orientation and theory	*What methodological orientation was stated to underpin the study? E.g. grounded theory, discourse analysis, ethnography, phenomenology, content analysis*<br>Thematic analysis	Materials and Methods/ Data analysis
Participant selection			
10.	Sampling	*How were participants selected? E.g. purposive, convenience, consecutive, snowball*<br>Convenience: FM individuals who declared to have experienced difficulties in the work environmentwere invited to participate.	Materials and Methods/Focus Group Study/Subjects enrolment
11.	Method of approach	*How were participants approached? E.g. face-to-face, telephone, mail, email*	Materials and Methods/Focus Group Study/Subjects enrolment

		Posts on the Facebook page and groups of CFU-Italia Odv. Then, FM sufferers communicated their interest in participating via email. These individuals were contacted by telephone to verify the eligibility criteria.	
12.	Sample size	*How many participants were in the study*? 15 (all females)	Results
13.	Non-participation	*How many people refused to participate or dropped out? What were the reasons for this?* No participant admitted to the study refused to participate or dropped out.	/
Setting			
14.	Setting of data collection	*Where was the data collected? E.g. home, clinic, workplace* Data for the 1st FG were collected in the headquarter of CFU-Italia Odv, in Castenaso. Data for the 2nd FG were collected in Rome, at an Italian Workers' Trade Unions Confederation offices.	Materials and Methods/Focus Group Study/Data collection
15.	Presence of non-participants	*Was anyone else present besides the participants and researchers?* One mother of a participant in Rome FG was present.	/
16.	Description of sample	*What are the important characteristics of the sample? E.g. demographic data, date* See "Focus Group results"	Results
Data collection			
17.	Interview guide	*Were questions, prompts, guides provided by the authors?* Yes *Was it pilot tested?* No	Supplementary material 2
18.	Repeat interviews	*Were repeat interviews carried out? If yes, how many?*	/

		N.A.	
19.	Audio/visual recording	*Did the research use audio or visual recording to collect the data?*<br>Yes, audio and video recorded	Materials and Methods/Focus Group Study/Data collection
20.	Field notes	*Were field notes made during and/or after the interview or focus group?*<br>Yes, during the FGs from the observer	/
21.	Duration	*What was the duration of the interviews or focus group?*<br>2 hours	Research Design and Methods/Data collection
22.	Data saturation	*Was data saturation discussed?*<br>The two FG provided similar results, no further FG were then organized taking also into account the extreme difficulties that FM sufferers encounters.	Method
23.	Transcripts returned	*Were transcripts returned to participants for comment and/or correction*? No	/
**Domain 3: analysis and findings**			
Data analysis			
24.	Number of data coders	*How many data coders coded the data?* Two data coders (MT and MP) and 1 reviewer (VT).	Research Design and Methods/Data analysis
25.	Description of the coding tree	*Did authors provide a description of the coding tree?* No, only the final results in terms of the themes/subthemes emerged is presented	Results/Table 5
26.	Derivation of themes	*Were themes identified in advance or derived from the data?* Both	Materials and Methods/ Data analysis and Table 5
27.	Software	*What software, if applicable, was used to manage the data?* No software was used; transcription were recorded in Microsoft Word.	/
28.	Participant checking	*Did participants provide feedback on the findings?*	/

		No, but an invitation to a free webinar on research results were sent to all participants and CFU-Italia Odv members.	
Reporting			
29.	Quotations presented	*Were participant quotations presented to illustrate the themes / findings?* Yes.<br><br>*Was each quotation identified? E.g. Participant number*<br><br>Quotations were not identified	Results
30.	Data and findings consistent	*Was there consistency between the data presented and the findings?*<br><br>Yes	/
31.	Clarity of major themes	Were major themes clearly presented in the findings?<br><br>Yes, each theme had a section	Results
32.	Clarity of minor themes	*Is there a description of diverse cases or discussion of minor themes*?<br><br>Yes, important unique opinions were taken into account together with specific sub-themes that inductively came out from the FGs	Research Design and Methods/Data analysis
